# Smartphone use and mental health: going beyond school restriction policies

**DOI:** 10.1016/j.lanepe.2025.101237

**Published:** 2025-02-08

**Authors:** Helen A. Weiss, Chris Bonell

**Affiliations:** a International Statistics and Epidemiology Group, London School of Hygiene & Tropical Medicine, London, UK; b Department of Public Health, Environments and Society, Faculty of Public Health and Policy, London School of Hygiene & Tropical Medicine, London, UK

Smartphone and social media use are plausibly associated with poorer mental health outcomes among adolescents, with likely mechanisms including: increased access to cyberbullying and views supportive of eating disorders, suicide, and self-harm^1^; impaired cognitive functions^2^; unhealthy social comparisons^3^; and displacement of healthy behaviours (sleep, exercise, in-person social interactions).^4^ However, the epidemiological evidence for a causal association is weak,^5^^,^^6^ as most studies are ecological and/or cross-sectional, showing correlation rather than causation and not adjusting for potential confounders.^6^ The associations between social media use and mental health are likely complex and bidirectional,^7^ and there is little causal evidence to date that reducing the time spent on social media will improve mental health and wellbeing.^6^

Despite the evidence-gap on the benefits of reduced social media time, governments are implementing or considering bans on smartphones in schools to improve adolescent wellbeing, mental health and educational outcomes. In February 2024, the UK Government issued non-statutory guidance that schools prohibit use of mobile phones throughout the school day (including breaks).^8^ In this issue of the Lancet Regional Health - Europe, Goodyear and colleagues report findings from the first study (“SMART Schools”) to rigorously evaluate the impact of the UK restrictive school phone policies on key health and educational outcomes.^9^ In this multi-method study, the authors used an observational study to compare mental wellbeing, sleep and educational outcomes among 1227 adolescents aged 12–15 years in 30 schools in the UK. Of these, 20 schools had implemented restrictive policies (recreational phone use not permitted, usually requiring phones to be kept switched off in school bags) and 10 schools had permissive policies (recreational phone use permitted at any time or at certain times and/or in certain places).

The study found no evidence that school phone policies were associated with improved mental wellbeing, anxiety, depression, problematic social media use, sleep health (duration, efficiency, timing), physical activity or educational attainment. As expected, participants in schools with restrictive policies reported less phone use during the school day (by about 30 min), but compensated for this when outside of school, so overall there was no evidence of a difference in screen time or social media time on weekdays or weekends. The study had a rigorous design and sampling strategy but was limited by a poor school response rate (10%), and the cross-sectional non-randomised design, which limits conclusions about causal effects of the restricted policy. The study did confirm findings from other cross-sectional studies, that students with longer duration of smartphone use or social media time had poorer wellbeing, mental health scores, sleep and educational attainment.^3^

The lack of impact of the restrictive phone policy on mental wellbeing may partly be because there was no reduction in screentime or social media use overall, only during school hours. Despite this lack of effects, phone bans in school might be an appropriate intervention to benefit school functioning and educational engagement. Future studies could look at outcomes such as school climate, bullying and social connectedness, and on improved memory, concentration and learning.

More generally, a focus on total screentime and social media use is likely to fail to address the diverse and complex mechanisms via which social media use might impact on mental health. Studies suggest that social comparison, passive versus active use of social media and various motives for social media use may have more influence on depression, anxiety and psychological distress than merely the duration of use.^6^^,^^10^

One lesson from this research is that to reduce social media use among adolescents, comprehensive measures are needed to address use at home as well as at school. The UK Government guidance on phone use in schools is unlikely to be sufficient to have an impact on poor mental health, wellbeing and education. However, improving smartphone habits at home is challenging, especially given the cognitive predilection of adolescents towards peer interactions and influence. We need improved parental education and guidance on how to reduce and improve their children's phone use, including use of parental control mechanisms, guidance on online safety, and understanding the importance of sleep and physical activity, and in-person socialising. Unfortunately, such interventions are likely to be least effective for those most in need or socially disadvantaged. We also need a regulatory approach to the technology industry that is flexible and evidence-based, focussing on preventing the forms of social media use that are most harmful, rather than merely focused on screentime limits. This is essential to optimise use of social media among adolescents in this time of rapid technological change.

This commentary was conceptualised and written by HAW and CB.

## Declaration of interests

We declare no competing interests.

## References

[1] Mento C., Silvestri M.C., Muscatello M.R.A. (2021). Psychological impact of pro-anorexia and pro-eating disorder websites on adolescent females: a systematic review. Int J Environ Res Public Health.

[2] Wacks Y., Weinstein A.M. (2021). Excessive smartphone use is associated with health problems in adolescents and young adults. Front Psychiatry.

[3] Keles B., McCrae N., Grealish A. (2020). A systematic review: the influence of social media on depression, anxiety and psychological distress in adolescents. Int J Adolesc Youth.

[4] Khalaf A.M., Alubied A.A., Khalaf A.M., Rifaey A.A. (2023). The impact of social media on the mental health of adolescents and young adults: a systematic review. Cureus.

[5] Stiglic N., Viner R.M. (2019). Effects of screentime on the health and well-being of children and adolescents: a systematic review of reviews. BMJ Open.

[6] Mansfield K.L., Ghai S., Hakman T., Ballou N., Vuorre M., Przybylski A.K. (2025). From social media to artificial intelligence: improving research on digital harms in youth. Lancet Child Adolesc Health.

[7] Hartanto A., Quek F.Y.X., Tng G.Y.Q., Yong J.C. (2021). Does social media use increase depressive symptoms? A reverse causation perspective. Front Psychiatry.

[8] Mobile phones in schools. Guidance for schools on prohibiting the use of mobile phones throughout the school day. https://assets.publishing.service.gov.uk/media/65cf5f2a4239310011b7b916/Mobile_phones_in_schools_guidance.pdf.

[9] Goodyear V. (2025). School phone policies, phone and social media use, and the association with mental health and wellbeing, sleep, physical activity, and educational outcomes: the SMART Schools cross sectional observational study. Lancet Reg Health Eur.

[10] Shannon H., Bush K., Villeneuve P.J., Hellemans K.G., Guimond S. (2022). Problematic social media use in adolescents and young adults: systematic review and meta-analysis. JMIR Ment Health.

